# Neogenin, a regulator of adult hippocampal neurogenesis, prevents depressive-like behavior

**DOI:** 10.1038/s41419-017-0019-2

**Published:** 2018-01-08

**Authors:** Dong Sun, Xiang-Dong Sun, Lu Zhao, Dae-Hoon Lee, Jin-Xia Hu, Fu-Lei Tang, Jin-Xiu Pan, Lin Mei, Xiao-Juan Zhu, Wen-Cheng Xiong

**Affiliations:** 1 Key Laboratory of Molecular Epigenetics of Ministry of Education, Institute of Cytology and Genetics, Northeast Normal University, Changchun, Jilin 130024 China; 2 Department of Neuroscience & Regenerative Medicine and Department of Neurology, Augusta University, Augusta, GA 30912 USA; 3 Department of Neurology, The affiliated hospital of Xuzhou Medical University, Xuzhou, Jiangsu Province 221002 China

## Abstract

Adult neurogenesis in hippocampal dentate gyrus (DG) is a complex, but precisely controlled process. Dysregulation of this event contributes to multiple neurological disorders, including major depression. Thus, it is of considerable interest to investigate how adult hippocampal neurogenesis is regulated. Here, we present evidence for neogenin, a multifunctional transmembrane receptor, to regulate adult mouse hippocampal neurogenesis. Loss of neogenin in adult neural stem cells (NSCs) or neural progenitor cells (NPCs) impaired NSCs/NPCs proliferation and neurogenesis, whereas increased their astrocytic differentiation. Mechanistic studies revealed a role for neogenin to positively regulate Gli1, a crucial downstream transcriptional factor of sonic hedgehog, and expression of Gli1 into neogenin depleted NSCs/NPCs restores their proliferation. Further morphological and functional studies showed additional abnormities, including reduced dendritic branches and spines, and impaired glutamatergic neuro-transmission, in neogenin-depleted new-born DG neurons; and mice with depletion of neogenin in NSCs/NPCs exhibited depressive-like behavior. These results thus demonstrate unrecognized functions of neogenin in adult hippocampal NSCs/NPCs-promoting NSCs/NPCs proliferation and neurogenesis and preventing astrogliogenesis and depressive-like behavior, and suggest neogenin regulation of Gli1 signaling as a possible underlying mechanism.

## Introduction

In adult mammalian brains, two neurogenic regions retain the ability to generate new neurons^1^. First, it is the subventricular zone (SVZ) of the lateral ventricular region, which generates neuroblasts that migrate into olfactory bulb (OB) through the rostral migratory stream and then differentiate into GABA and dopamine-producing interneurons^2,3^. Second, it is the subgranular zone of dental gyrus (DG) of hippocampus, which gives rise to progenitors that undergo several developmental stages defined by specific markers and finally become local excitatory granule neurons^4,5^. Mature granule neurons can integrate into local circuitry and perform corresponding functions^6^. Reduction of adult neurogenesis impairs synaptic transmission and plasticity in the DG, and both long-term potentiation and long-term depression are regulated by adult neurogenesis^7^. Furthermore, several studies demonstrate that defective adult hippocampal neurogenesis contributes to depression^8,9^.

Adult neurogenesis is tightly controlled by cell intrinsic factors as well as local microenvironment, including transcription factors, growth factors, neuropeptides, and neurotransmitters. Among the intrinsic factors, Tlx, Sox2 and miR-124 are well known in regulating the proliferation of neural stem cells/neural progenitor cells (NSCs/NPCs)^10–12^. In addition, sonic hedgehog (Shh), Wnt/β-catenin, and Notch gene homolog2 (Notch2) pathways have been studied extensively for their functions in maintenance and self-renewal of NSCs/NPCs^13–15^. We are all aware that the SGZ is enriched in exuberant neuronal communications between mature granule neurons and inhibitory interneurons^16^, major neurotransmitters like Glutamate and GABA have long been implicated in regulating adult hippocampal neurogenesis^17,18^. Recently, mature granule neurons were also thought to compete with new-born neurons for synaptic inputs, and further affected NSCs activation^19^.

Neogenin, a member of deleted in colorectal cancer-family transmembrane proteins, appears to be a receptor or co-receptor for multi-ligands, including netrins, repulsive guidance molecules, and bone morphogenetic proteins (BMPs)^20–22^. Thus, it is implicated in various cellular functions in the brain, such as axon guidance, neuronal differentiation, neural tube formation, neuronal survival^21,23–25^, adult SVZ-OB neurogenesis^26^, and olfactory epithelium development^27^. In addition to the brain, neogenin plays important roles in non-brain organs, such as endochondral bone formation^28^, digit patterning^29^, and ion hemostasis^30,31^. Notice that neogenin is highly expressed in NSCs/NPCs, however, its function in adult hippocampal neurogenesis remains largely unknown.

Here, we provide evidence for neogenin’s function in regulation of adult mouse hippocampal neurogenesis as well as depressive-like behavior. Neogenin is highly expressed in NSCs/NPCs and immature neurons. Selective depletion of neogenin in hippocampal NSCs/NPCs impaired their proliferation and neurogenesis, but increased their astrocytic differentiation. Further analysis showed that neogenin-deficient new-born neurons in DG displayed multiple abnormities, including reduced dendritic branches and spines and impaired glutamatergic neuro-transmission. In addition, mice with depletion of neogenin in NSCs/NPCs showed depressive-like behavior, supporting the view for adult hippocampal neurogenesis to be crucial in hippocampus-dependent mood regulation. Together, these results demonstrate critical functions of neogenin in promoting adult hippocampal neurogenesis and preventing depressive-like behavior.

## Results

### Neogenin expression in NSCs/NPCs and immature neurons in adult mouse hippocampal DG

**Fig. 1:**
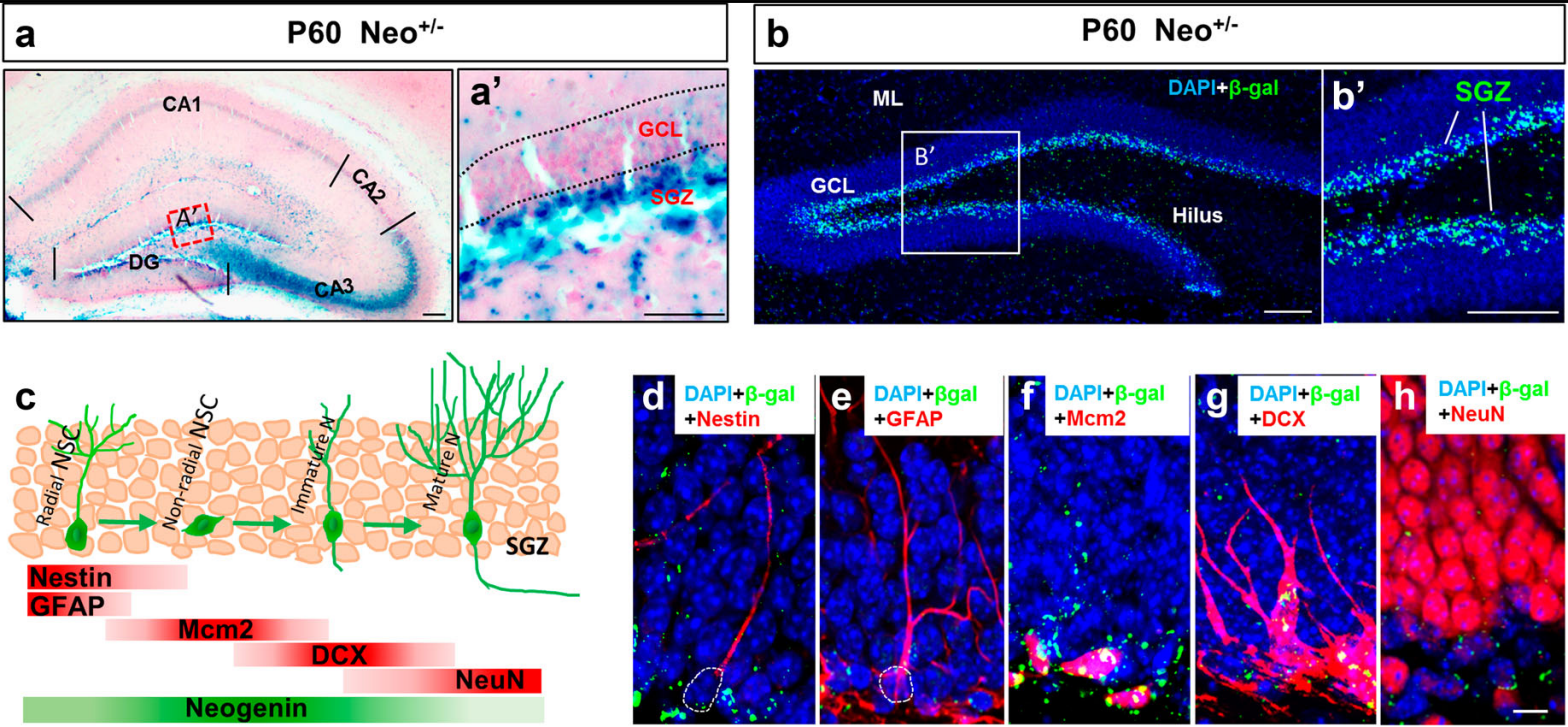
Neogenin expression in NSCs/NPCs and immature neurons in adult mouse hippocampal dentate gyrus **a** X-gal staining (blue) analysis for *lacZ* gene expression (neogenin) in 2-month-old Neo^+/−^ brain sections. **a’** a high magnification of the select region in **a**. Scale bars: 100μm in **a** and 50μm in **a’**. **b** Immunostaining of β-gal (green) in 2-month-old mouse hippocampal DG. The select region was amplified and shown in **b’**. Scale bars: 100 μm in **b** and **b’**. **c** Schematic illustration of various types of cells in adult mouse hippocampal SGZ. **d–h** Co-immunostaining analyses of β-gal (green) with different cell markers: Nestin or GFAP for radial NSCs; Mcm2 for non-radial NSCs; DCX for immature neurons, and NeuN for mature neurons. Scale bars: 10 μm in **d**–**h**

To investigate neogenin’s function in adult hippocampus, we first examined its expression by taking advantage of *LacZ* reporter in neogenin mutant mice. The *LacZ* gene is knocked in the intron of neogenin gene, thus, the *LacZ* activity, under the control of neogenin promoter, has been used as a reporter for neogenin’s expression^28,31–33^. The LacZ enzymatic activity by X-gal staining was strong in regions of subgranule zone (SGZ) of dentate gyrus (DG) and CA3, but weak in CA1 and CA2 zones (Fig. 1a and a’). The high level of LacZ expression in SGZ was further confirmed by immunostaining analysis using anti-β-gal antibody (Fig. 1b and b’). As SGZ region contains various types of cells, including Nestin^+^ or GFAP^+^ radial glia-like NSCs, Mcm2^+^ non-radial NSCs (or transient amplifying NSCs/NPCs), glial cells (e.g., astrocytes and microglia), and DCX^+^ immature neurons (Fig. 1c), we asked in which cell type(s) in the SGZ that neogenin is expressed. Co-immunostaining analysis showed that neogenin’s β-gal reporter was detected in Nestin^+^ or GFAP^+^ radial glia-like NSCs (Fig. 1d, e), but enriched in Mcm2^+^ transient amplifying NSCs/NPCs, as well as DCX^+^ immature neurons (Fig. 1f, g). The β-gal signal was barely detected in the granule cell layer (GCL) where NeuN^+^ mature neurons reside (Fig. 1h), and un-detectable in microglia (data not shown). These results thus demonstrate abundant neogenin’s expression in NSCs/NPCs and immature neurons in the SGZ (Fig. 1c), implicating neogenin’s function in adult hippocampal neurogenesis.

### Impaired adult hippocampal neurogenesis in mice that selectively knocked out neogenin in NSCs/NPCs

To investigate neogenin’s function in NSCs/NPCs, we generated *Neo*^*f/f*^*;Gli1-CreER*^*T2*^*;Ai9* (*Neo*^*Gli1CreER*^-CKO) mice by crossing floxed neogenin (*Neo*^*f/f*^) with *Gli1-CreER*^*T2*^*;Ai9* mice (Supplementary Figs. S1A, B, S2A, B) for the following reasons. First, neogenin’s expression in SGZ resembles to that of Gli1 (GLI-Kruppel family member), a transcription factor downstream of Shh signaling that has been used as a sensitive readout of Shh activity to label Shh-responding cells in adult hippocampus^34^. Second, the expression of Cre in SGZ of *Gli1-CreER*^*T2*^ mice (inducible Cre recombinase expression driven by Gli1 promotor) was verified by crossing *Ai9* mice with *Gli1-CreER*^*T2*^ to get *Gli1-CreER*^*T2*^*;Ai9* mice (Supplementary Fig. S1A, B). Upon tamoxifen injection, *Gli1-CreER*^*T*2^ is activated to mediate efficient recombination in Ai9, and the tdTomato is thus turned on to represent Shh-responding NSCs and their progenies (Supplementary Fig. S1C). As reported^34–36^, Nestin^+^ or GFAP^+^ radial glia-like NSCs were positive for tdTomato in *Gli1-CreER*^*T2*^*;Ai9* mice treated with tamoxifen for 7 days (D) (Supplementary Fig. S1D, E). The tdTomato was also detected in Mcm2^+^ transient amplifying NSCs/NPCs and in DCX^+^ immature neurons (Supplementary Fig. S1F, G). These results confirmed the expression of Gli1-Cre in NSCs/NPCs and immature neurons in the SGZ (Supplementary Fig. S1H), demonstrating a similar expression pattern of Gli1-Cre to that of neogenin. Finally, the endogenous neogenin was efficiently deleted in DG of *Neo*^*Gli1CreER*^-CKO mice, compared to that of control mice (Supplementary Fig. S2C–E).

**Fig. 2:**
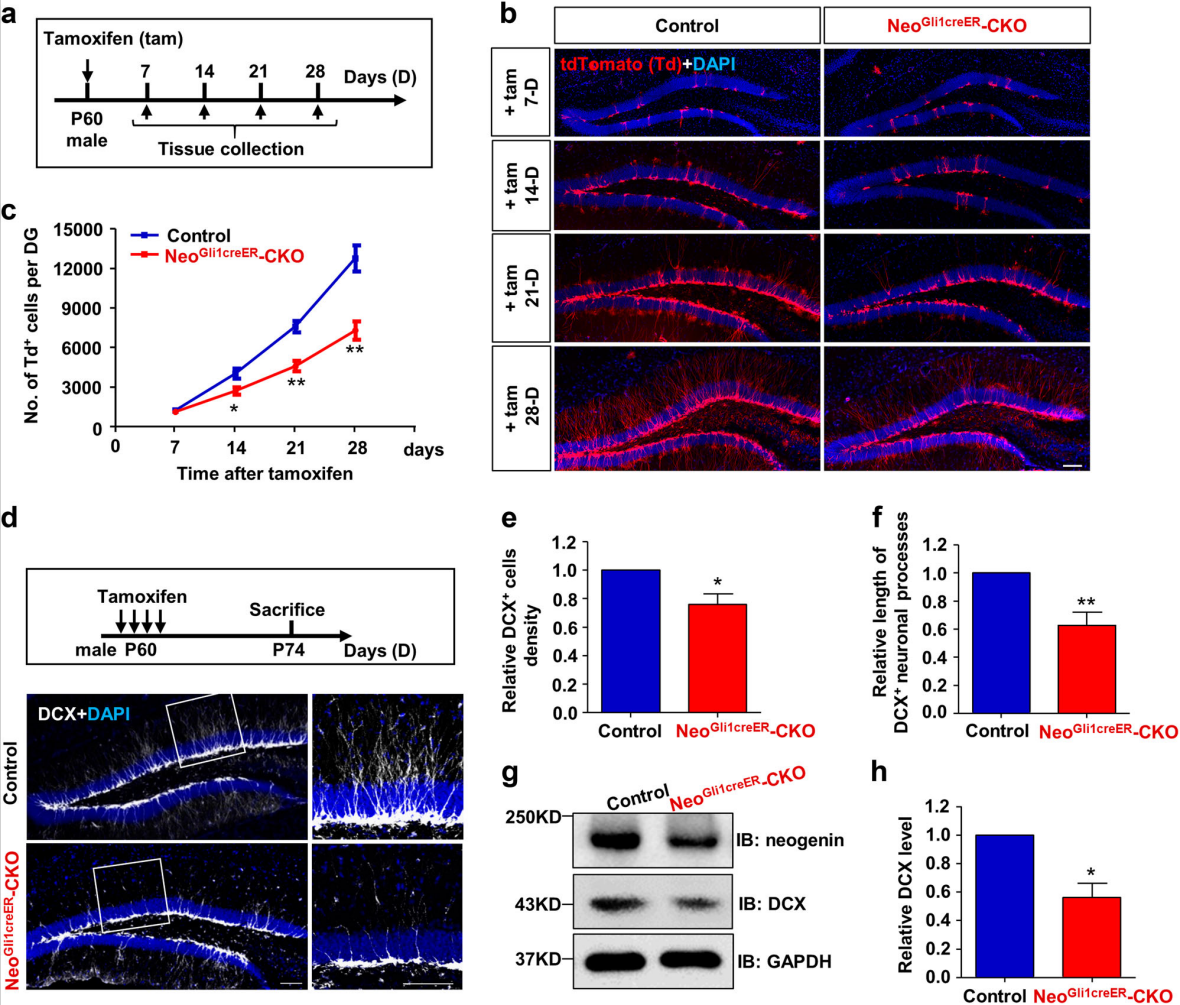
Reduced adult hippocampal neurogenesis in *Neo*^*Gli1CreER*^-CKO mice **a** Schematic diagram of tamoxifen treatment and tissue (hippocampus) collection at indicated times after tamoxifen treatment. **b** Immunostaining analysis of tdTomato (red) at indicated times in the hippocampus of control (*Neo*^*+/+*^*;Gli1-CreER*^*T2*^*;Ai9*) and *Neo*^*Gli1CreER*^-CKO mice. Scale bar = 100 μm. **c** Quantitative analysis of the numbers of tdTomato^+^ cells per DG. Data presented are mean ± SEM. (*n* = 5 per genotype). *P < 0.05; **P < 0.01. Student’s *t*-test. **d** Experimental scheme of tamoxifen injections and immunostaining analysis of DCX (white) in hippocampus of control and *Neo*^*Gli1CreER*^-CKO mice. Scale bars = 100 μm. **e** Quantitative analysis of relative DCX^+^ cell density in **d**. **f** Quantitative analysis of relative process length of DCX^+^ neurons in **d**. **g-h** Western blot analysis of DCX protein levels in control and *Neo*^*Gli1CreER*^-CKO hippocampus. Representative blots were shown in **g**, and quantification of the relative DCX protein levels was presented in **h**. Data in **e**, **f**, and **h** are presented as the mean ± SEM (*n* = 3 per genotype). *P < 0.05; **P < 0.01. Student’s *t*-test

We next investigated neogenin’s function in SGZ cells by comparing the numbers of tdTomato^+^ cells between control (*Neo*^*+/+*^*;Gli1-CreER*^*T2*^*;Ai9*) and *Neo*^*Gli1CreER*^-CKO mice injected with tamoxifen at age of P60 (postnatal day 60) for 7, 14, 21 and 28-days, which are critical developmental stages for adult new-born neurons development (Fig. 2a). Whereas control mice showed time-dependent increases of tdTomato^+^ cells, *Neo*^*Gli1CreER*^-CKO mice displayed marked decreases in 14, 21, 28 days, but not 7 days, after tamoxifen injection (Fig. 2b, c). These results implicate neogenin in neurogenesis.

We then asked whether DCX^+^ immature neurons were reduced in *Neo*^*Gli1CreER*^-CKO mice. Both control and mutant mice were injected with tamoxifen at age of P60 for four times (125 mg/kg/time, 1 time/12 h) to achieve higher efficiency, and 14 days after tamoxifen treatment, DCX^+^ immature neurons were examined by immunostaining and Western blot analyses (Fig. 2d–h). Consistent with the result of decreased tdTomato^+^ cells, DCX^+^ immature neuronal density was significantly lower in *Neo*^*Gli1CreER*^-CKO mice than that of control mice (Fig. 2d, e). In supporting this view, the DCX protein levels were reduced in *Neo*^*Gli1CreER*^-CKO hippocampus (Fig. 2g, h). The reduction in tdTomato^+^ or DCX^+^ cells was not due to an increased apoptosis, as cleaved-caspase3 (c-caspase3), a marker for apoptosis, was not increased in the mutant DG (Supplementary Fig. S3). Further analysis of DCX^+^ neuronal morphology showed a much shorter dendritic process in the mutant DG (Fig. 2d, f), suggesting that in addition to the genesis of these immature neurons, neogenin also regulates their dendritic growth.

In addition to NSCs/NPCs in SGZ, neogenin is expressed in CA3 neurons and GFAP^+^ astrocytes (Fig. 1 and data not shown). We thus asked whether neogenin expression in these cells plays a role in adult hippocampal neurogenesis. To this end, *Neo*^*NestinCreER*^-CKO, *Neo*^*GFAPCreER*^-CKO, and *Neo*^*Nex*^*-*CKO mice were generated by crossing *Neo*^*f/f*^ with *Nestin-CreER*, *GFAP-CreER*, and *Nex-Cre* mice, respectively. Injections with tamoxifen in P60 old *Neo*^*NestinCreER*^-CKO and *Neo*^*GFAPCreER*^-CKO mice for 14 days (Supplementary Fig. S4A) resulted in selective knocking out of neogenin in NSCs and astrocytes, respectively (data not shown). *Neo*^*Nex*^*-*CKO selectively knocked out neogenin in pyramidal neurons^32^. As shown in Supplementary Fig. S4B–D, DCX^+^ immature neurons and their processes were markedly reduced in *Neo*^*NestinCreER*^-CKO mice, compared with that of control mice, in agreement with the results in *Neo*^*Gli1CreER*^-CKO mice. Interestingly, the reduction of DCX^+^ immature neurons was not observed in *Neo*^*GFAPCreER*^-CKO (Supplementary Fig. S4E–G), nor *Neo*^*Nex*^*-*CKO mice (Supplementary Fig. S4H–J), compared to their control mice. In contrast, an increase in DCX^+^ neurons was detected in *Neo*^*GFAPCreER*^-CKO mice (Supplementary Fig. S4E–G). These results suggest that neogenin expression in NSCs/NPCs, but not astrocytes or neurons, promotes genesis of DCX^+^ neurons.

### Decreased proliferation, but increased astrocytic differentiation, in neogenin-deficient NSCs/NPCs

**Fig. 3:**
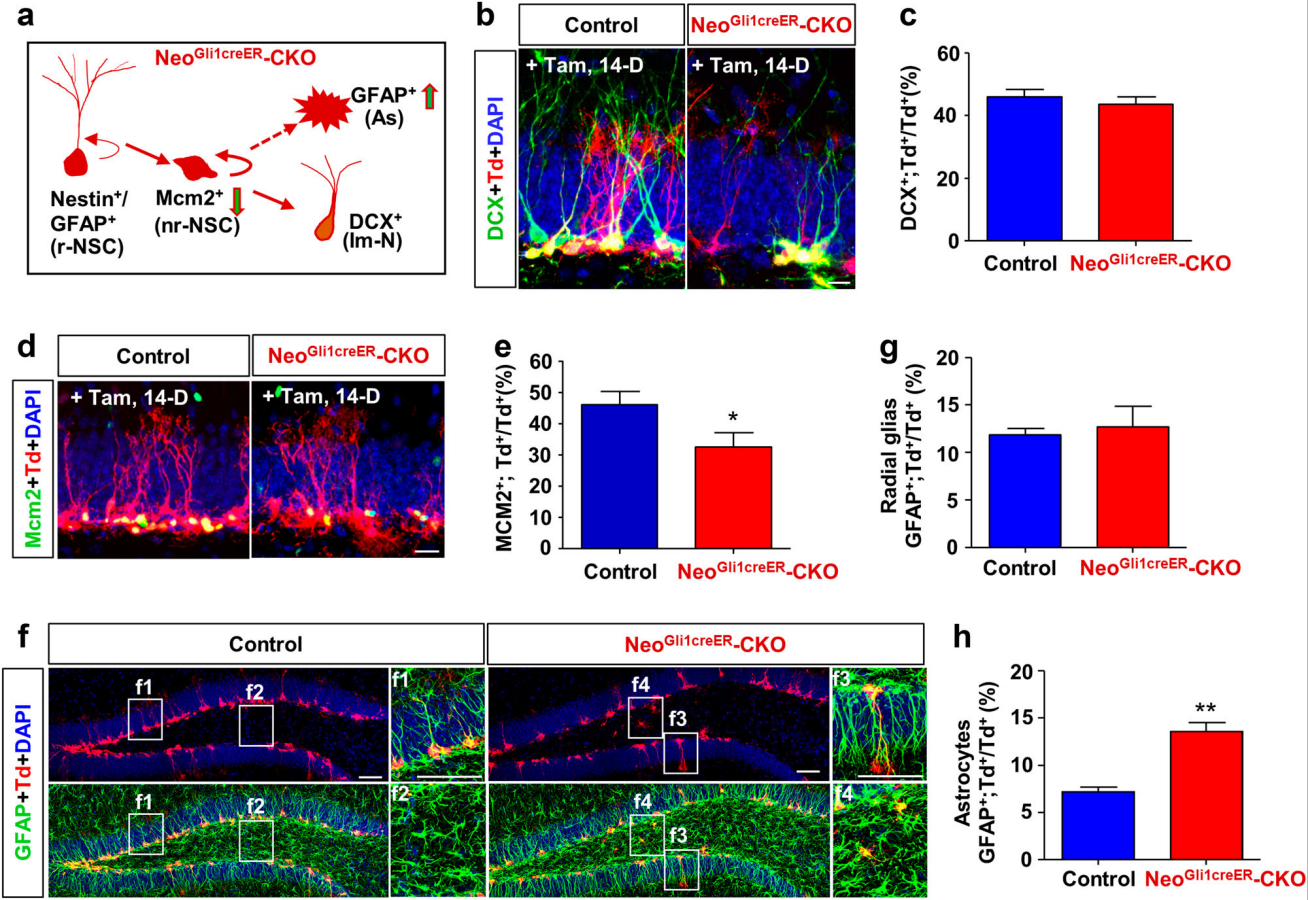
Decreased proliferation, whereas increased astrocytic differentiation, and no effect of neuronal differentiation from neogenin-deficient hippocampal NSCs/NPCs **a** Model of the proliferation and differentiation of hippocampal NSCs/NPCs. **b** Double immunostaining of tdTomato (red) and DCX (green) in hippocampus of control and *Neo*^*Gli1CreER*^-CKO mice. Scale bars = 50 μm. **c** Quantification of the ratio of tdTomato^+^; DCX^+^ over total tdTomato^+^ cells. **d** Double immunostaining of tdTomato (red) and Mcm2 (green). Scale bars = 50 μm. **e** Quantification of the ratio of tdTomato^+^ Mcm2^+^ over total tdTomato^+^ cells. **f** Double immunostaining analysis of tdTomato (red) and GFAP (green). f1–f4 showed a higher magnification of the selected regions. Radial glia-like NSCs (f1 and f3): Bipolar shape with a long radial process, located in SGZ of DG. Astrocytes (f4): stellate shape with star-like short processes, located in SGZ, granule cell layer and hilus. Scale bars = 50 μm. **g**, **h** Quantification of the radial glia-like NSCs **g** or astrocytes **h** ratio of tdTomato^+^; GFAP^+^ over total tdTomato^+^ cells. Data are presented as the mean ± SEM. (*n* ≥ 500 cells). *P < 0.05; **P < 0.01. Student’s *t*-test

DCX^+^ immature neurons are derived from transient amplifying NSCs/NPCs^37^. These NSCs/NPCs, marked by Mcm2, are proliferative, and from nestin^+^ or GFAP^+^ radial glia-like NSCs^38^ (Fig. 3a). To understand how neogenin in NSCs/NPCs promotes DCX^+^ neurogenesis, we thus co-immunostained tdTomato with DCX, Mcm2, or GFAP antibodies in control and *Neo*^*Gli1CreER*^-CKO mice injected with tamoxifen at P60 for 14 days (Fig. 3b, d, f). To our surprise, the percentage of DCX and tdTomato double positive cells over total tdTomato^+^ cells was unaffected in *Neo*^*Gli1CreER*^-CKO mice (Fig. 3b, c); however, the percentage of Mcm2 and tdTomato double positive cells over total tdTomato^+^ cells was significantly lower in the mutant mice than that of controls (Fig. 3d, e). In contrast, the percentage of GFAP and tdTomato double positive astrocytes over total tdTomato^+^ cells was elevated in the mutant mice, but the GFAP^+^ radial glia-like NSCs were unaffected (Fig. 3f–h). These results implicate neogenin’s dual functions in Mcm2^+^ NSCs/NPCs: a positive role for Mcm2^+^ NSCs/NPCs proliferation and a negative role for astrogliogenesis.

**Fig. 4:**
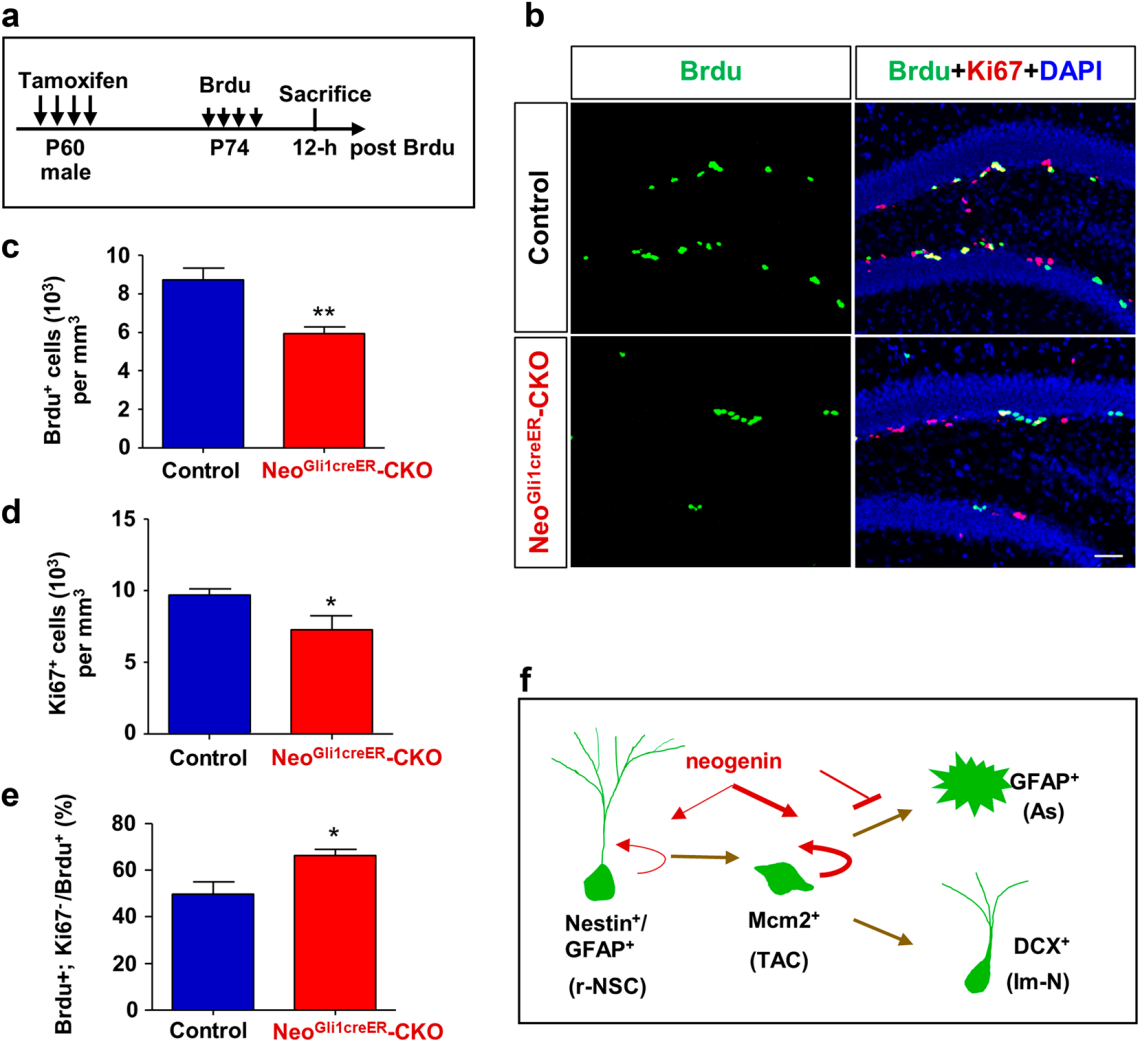
Reduced proliferation, and promoted their cell cycle exit, from neogenin deficient hippocampal NSCs/NPCs **a** Schematic diagram of multiple tamoxifen and Brdu injections for indicated time. **b** Double immunostaining of Brdu (green) and Ki67 (red) in hippocampus of control and *Neo*^*Gli1CreER*^-CKO mice. Scale bars = 100μm. **c**, **d** Quantification of Brdu^+^ cell density in **c** and Ki67^+^ cell density in **d**. Data are mean ± SEM. (*n* = 5 per genotype). *P < 0.05; *P < 0.01. Student’s *t*-test. **e** Quantification of the ratio of Brdu^+^; Ki67^-^ over total Brdu^+^ cells. Data are mean ± SEM. (*n* ≥ 500 cells). *P < 0.05. Student’s *t*-test. **f** Model of neogenin promotion of NSC/NPC proliferation, but suppression of their astrocytic differentiation

To test this view, Brdu, a marker for cell proliferation, was injected into tamoxifen treated control and mutant mice, and 12 h after Brdu injection, mice were sacrificed for analysis (Fig. 4a). As shown in Fig. 4b, c, neogenin deletion resulted in a significant reduction of Brdu^+^ cells in SGZ, supporting the view for a positive role of neogenin in NSC/NPC proliferation. This view was further supported by co-immunostaining analysis of Ki67 (an endogenous mitotic marker for cell proliferation), which showed decreased Ki67^+^ cells in *Neo*^*Gli1CreER*^-CKO mice, compared with that of control mice (Fig. 4d). Notice that the percentage of Brdu^+^ and Ki67^-^ cells over total Brdu^+^ cells was higher in *Neo*^*Gli1CreER*^-CKO mice than that of controls (Fig. 4e), suggesting that more Brdu^+^ NSCs/NPCs undergone cell cycle exit or had completed the cell cycle when neogenin was depleted, which supported the view for neogenin in promoting NSCs/NPCs proliferation, but suppressing their astrocytic differentiation (Fig. 4f).

**Fig. 5:**
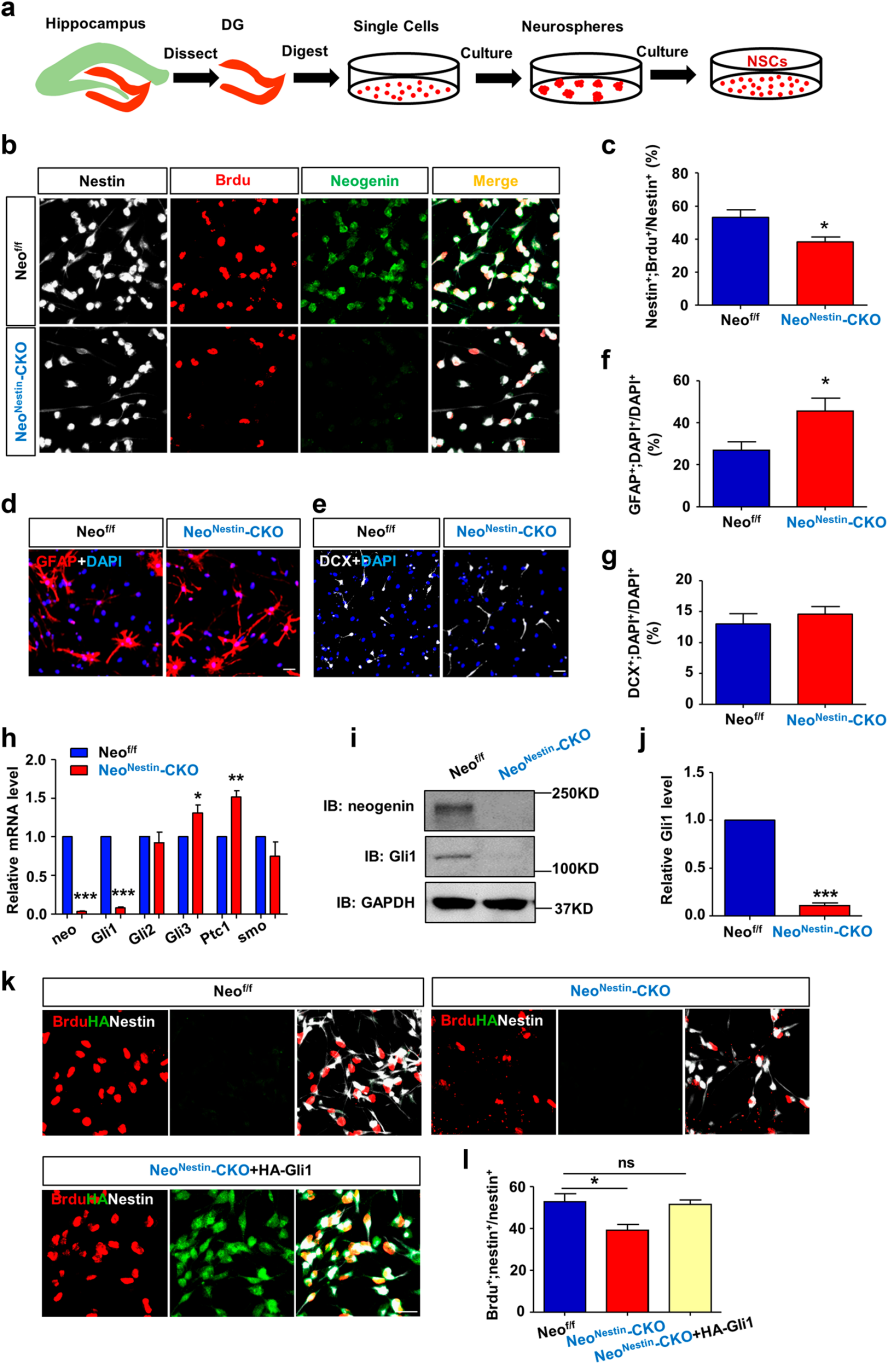
Loss of neogenin reduced Gli1-mediated proliferation, but promoted astrocytic differentiation, in cultured NSCs **a** Schematic drawing shows the isolation of DG-NSCs. **b** Brdu analysis for NSCs proliferation in vitro. Nestin (red), Brdu (white) and neogenin (green). (*n* ≥ 1000 cells from 5 slides). Scale bars = 50μm. **c** Quantification of Brdu incorporated ratio of Brdu^+^;Nestin^+^ over total Nestin^+^ cells. **d**, **e** Immunostaining of GFAP **d** or DCX **e** in differentiated cells in vitro. (*n* ≥ 800 cells from 5 slides). Scale bars = 20μm. **f**, **g** Quantification of the ratio of GFAP^+^**f** or DCX^+^**g** cells over total cells. **h** Real-time PCR (RT-PCR) analysis of relative gene levels of Shh signaling pathway (Normalized to NSCs of *Neo*^*f/f*^ mice). **i** Western blot analysis of Gli1 protein levels in *Neo*^*f/f*^ and *Neo*^*Nestin*^-CKO NSCs. **j** Quantification of the relative Gli1 protein levels in I. (*n* = 3 per genotpye) **k** Brdu analysis for NSCs proliferation in three groups (Neo^f/f^, Neo^Nestin^-CKO and Neo^Nestin^-CKO + Gli1). Scale bars = 10μm. **l** Quantification of the Brdu incorporated ratio, Brdu^+^;Nestin^+^ over total Nestin^+^ cells. Data are presented as the mean ± SEM. (*n* ≥ 1000 cells from 5 slides). *P < 0.05; **P < 0.01; *** *P* < 0.001. Student’s *t*-test or one-way ANOVA plus post-hoc analysis

To further examine neogenin’s function in NSCs/NPCs proliferation, hippocampal NSCs were isolated from 2-month old *Neo*^*f/f*^ and *Neo*^*Nestin*^*-*CKO mice (Fig. 5a). As expected, endogenous neogenin was much lower in *Neo*^*Nestin*^*-*CKO NSCs than that of control NSCs (Supplementary Fig. S5A). The sizes of neurospheres were smaller in the mutant NSCs than those of controls, implicating a reduced growth of mutant NSCs (Supplementary Fig. S5B and S5C). We then planted NSCs on poly-L-ornithine and laminin-coated coverslips, and cultured with proliferation medium (Neurobasal medium + B27 + GlutaMAX + FGF-2 + EGF) containing Brdu (5 μM) for 6 h. The Brdu incorporation in *Neo*^*Nestin*^*-*CKO NSCs was markedly reduced, compared with that of controls (Fig. 5b, c). Taken together, these results provide in vitro and in vivo evidence for neogenin’s function in promoting NSCs/NPCs proliferation.

We next examined neogenin’s function in astrocytic and neural differentiation from NSCs in vitro. The isolated NSCs from *Neo*^*f/f*^ and *Neo*^*Nestin*^*-*CKO mice were planted onto poly-L-ornithine and laminin-coated coverslips and cultured with the differentiation medium (Neurobasal medium + B27 + GlutaMAX) for 3 days. Cells were fixed and immunostained with GFAP and DCX antibodies to label astrocytes and neurons, respectively (Fig. 5d, e). In line with the results in vivo, neogenin-depleted NSCs displayed increased GFAP^+^ astrocytes, but a similar number of DCX^+^ neurons, compared with those of control cells (Fig. 5f, g). These results thus provide additional evidence for neogenin’s function in hippocampal NSCs to suppress astrocytic, but not neural, differentiation.

### Neogenin induction of Gli1 in hippocampal NSCs/NPCs to promote their proliferation

Shh signaling pathway is known to be essential for sustaining the proliferation of NSCs/NPCs in hippocampus^13^. We thus wonder whether neogenin regulates Shh pathway in hippocampal NSCs/NPCs to promote their proliferation. To test this view, we extracted mRNAs of hippocampal NSCs from *Neo*^*f/f*^ and *Neo*^*Nestin*^*-*CKO mice. Real time-PCR analyses of genes in the Shh pathway, including Gli1-3, patched1 (Ptc1), and smoothen (smo), were examined. Intriguingly, Gli1, a transcriptional factor downstream of Shh pathway and a sensitive readout of Shh activity, was significantly lower in *Neo*^*Nestin*^*-*CKO NSCs than that of *Neo*^*f/f*^ NSCs (Fig. 5h). In contrast, the mRNA levels of Gli3 and Ptc1 were increased in neogenin deficient NSCs (Fig. 5h). The reduction of Gli1 in *Neo*^*Nestin*^*-*CKO NSCs was further confirmed by Western blot analysis (Fig. 5i, j). We then asked if the reduced Gli1 in neogenin deficient NSCs is responsible for the impaired proliferation. To this end, neogenin deficient NSCs infected with or without retroviruses expressing HA-tagged Gli1 were cultured for 3-days, and incubated with the proliferation medium containing 5 μM Brdu for 6 h. Remarkably, expression of Gli1 in *Neo*^*Nestin*^*-*CKO NSCs showed a comparable level of Brdu incorporation to that of *Neo*^*f/f*^ NSCs (Fig. 5k, l), suggesting a critical role of Gli1 in mediating neogenin-promoted NSCs proliferation.

### Impaired dendritic morphogenesis and maturation in neogenin depleted adult new-born DG neurons

**Fig. 6:**
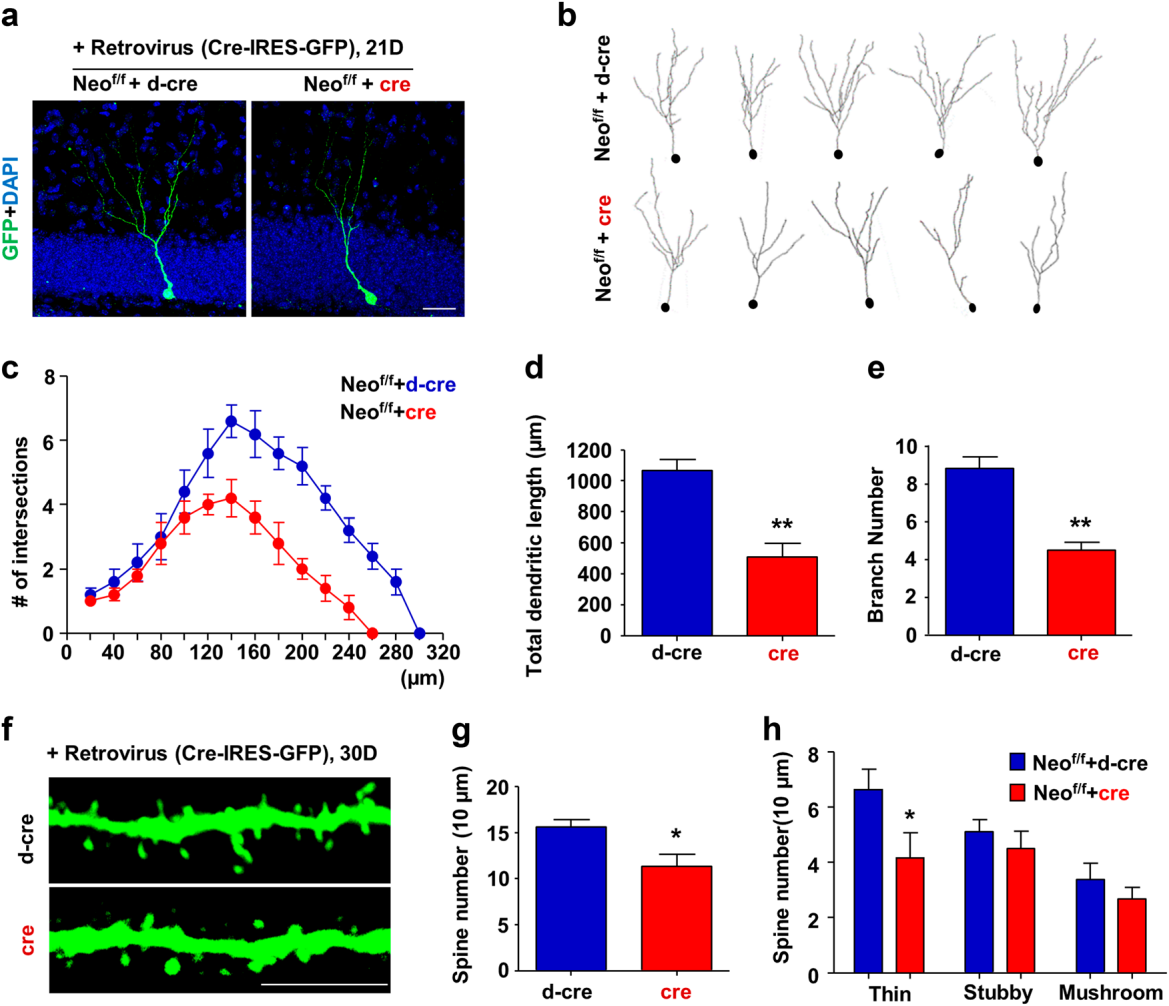
Impaired dendritic morphogenesis in neogenin deficient new-born neurons **a** Retroviruses expressing d-cre or cre were injected into DG of *Neo*^*f/f*^ mice for 21days. Scale bars = 20 μm. **b** Tracing of representative new-born neurons in the DG from both groups. **c** Sholl analysis of the dendritic complexity in **b** by ImageJ software. (*n* ≥ 50 cells). **d** Quantification of the dendritic total length. (*n* ≥ 50 cells). **e** Quantification of the branch number. (*n* ≥ 50 cells). **f** Representative spine images of neurons infected with retrovirus expressing d-cre or cre for 1-month. Scale bars = 5 μm. **g** Quantitative analysis of the number of spines in d-cre or cre expressing new-born neurons. **h** Quantitative analysis of different types of spines in d-cre or cre expressing new-born neurons. Data are mean ± SEM. (*n* ≥ 30 cells). *P < 0.05; **P < 0.01. Student’s *t*-test

Notice that in addition to the reduction of DCX^+^ neuronal density, their dendritic processes were much shorter in the mutant SGZ (Fig. 2d–f), implicating neogenin in promoting new born DG neuronal dendritic morphogenesis and maturation. We further tested this view by use of retrovirus driven Cre strategy, as retrovirus targets only dividing cells and labels their subsequent progenies^39^. Retroviruses encoding Cre or d-Cre (an inactive Cre, as a control) with IRES-EGFP (under the control of CAG promoter) were injected into the DG of *Neo*^*f/f*^ mice to infect NSCs/NPCs, and 3 weeks later, GFP-marked DG neurons were examined (Fig. 6a). To accurately acquire the three-dimensional image, each final image was composed of ten serial *z*-plane images by Zeiss confocal system. Indeed, the dendritic complexity was considerably reduced in Cre expressing (neogenin-CKO) new born neurons, compared with that of controls assessed by sholl analysis (Fig. 6b, c). Neogenin deficient new born neurons had markedly decreased total dendritic length and the number of dendritic branches based on morphologic analysis (Fig. 6d, e). High power imaging analysis of dendritic spines showed reduced spine density, particularly thin type of spines, in these new-born DG neurons from Cre expressing mice, compared with that of controls (Fig. 6f–h). These results suggest that neogenin in NSCs/NPCs or immature neurons plays a role in promoting new-born neuronal dendritic morphogenesis and maturation.

We next tested this view in control and *Neo*^*Gli1CreER*^-CKO mice, which were injected with tamoxifen at P45 for 1 month (Supplementary Fig. S6A). The tdTomato and NeuN double positive neurons (as new born DG neurons) were examined (Supplementary Fig. S6B). Again, dendritic complexity, length, and arborization were all reduced in *Neo*^*Gli1CreER*^-CKO mice (Supplementary Fig. S6C–F). Similar to the results described in Fig. 6, the dendritic spine density and thin spines were largely decreased in neogenin depleted neurons (Supplementary Fig. S6G–I). These results thus confirmed neogenin’s function in new born DG neuronal dendritic morphogenesis.

### Impairment of excitatory synaptic transmission and depressive-like behavior in mice that selectively knocked out neogenin in NSCs/NPCs

**Fig. 7:**
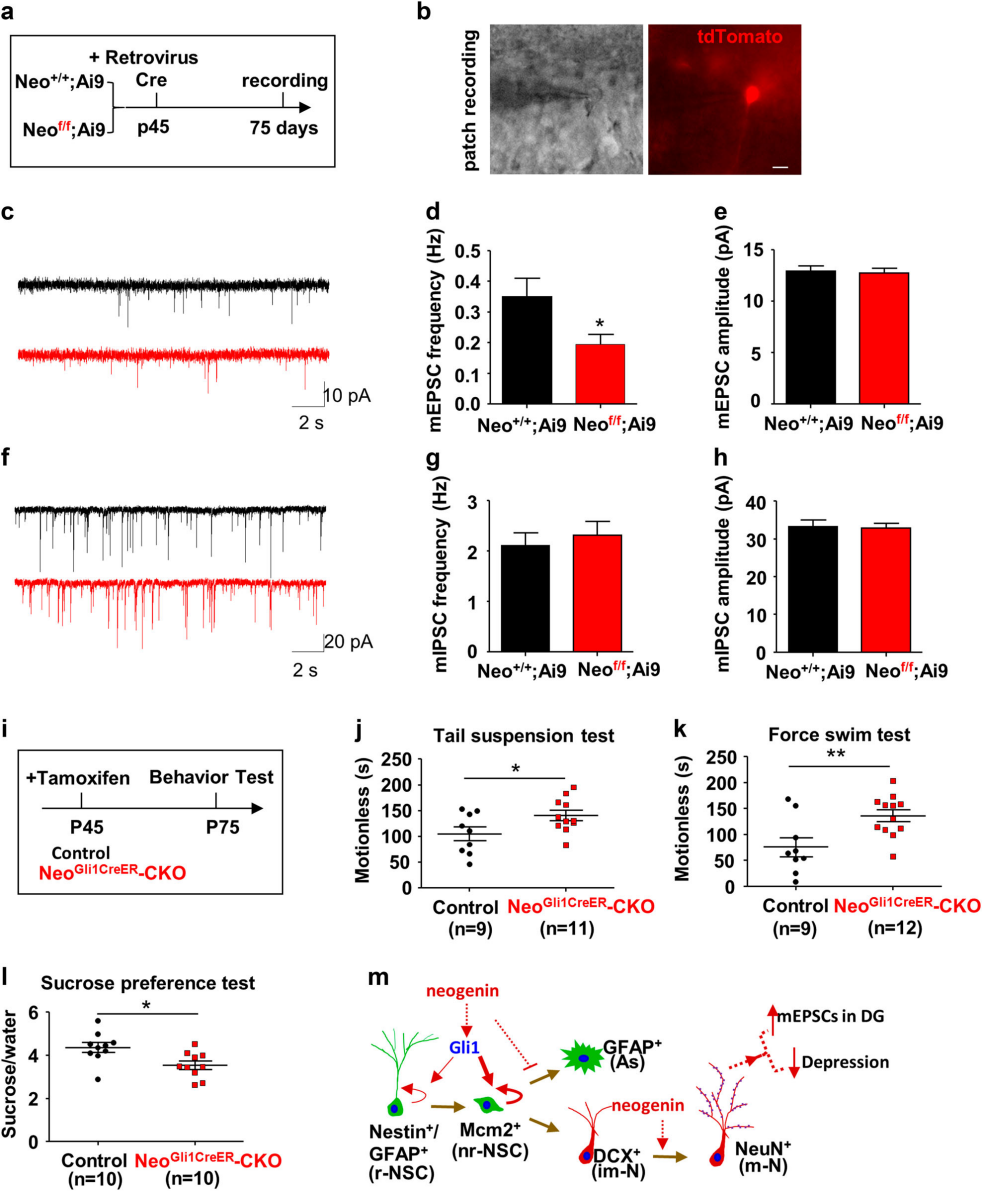
Impairment of excitatory synaptic transmission and depressive-like behavior in mice that selectively knocked out neogenin in NSCs/NPCs **a** Schematic drawing showed the strategy for electrophysiological recording. **b** Recording of tdTomato^+^ DG new-born neurons (red) in both *Neo*^*+/+*^*;Ai9* (control) and *Neo*^*f/f*^*;Ai9* (knockout neogenin) mice, Scale bars = 10 μm. **c** Representative traces of mEPSC of new-born neurons from *Neo*^*+/+*^*;Ai9* and *Neo*^*f/f*^*;Ai9* mice. **d**, **e** Quantitative analysis of the frequency **d** and amplitude **e** of mEPSC. (*n* ≥ 10 neurons). **f** Representative traces of mIPSC of new-born neurons from *Neo*^*+/+*^*;Ai9* and *Neo*^*f/f*^*;Ai9* mice. **g**, **h** Quantitative analysis of the frequency **g** and amplitude **h** of mIPSC. (*n* ≥ 10 neurons). **i** Schematic diagram of tamoxifen treatment and behavior test. **j**, **k** Control and *Neo*^*Gli1CreER*^-CKO mice were subjected to the TST **j** and FST **k**, and quantification of the immobility time in both groups. **l** Control and *Neo*^*Gli1CreER*^-CKO mice were subjected to sucrose preference test. The ratio of sucrose verse water consumed was quantified. Data are presented as the mean ± SEM. (*n* ≥ 9 per genotype). *P < 0.05; **P < 0.01. Student’s *t*-test. (**m**) Model of neogenin’s functions in adult hippocampal neurogenesis

We next asked whether loss of neogenin has functional deficit in their synaptic transmission. To this end, the retrovirus-expressing Cre was injected into hippocampus to infect NSCs at DG of *Neo*^*+/+*^*;Ai9* or *Neo*^*f/f*^*;Ai9* mice for 1 month; and new-born neurons expressing tdTomato were used for electrophysiological recording (Fig. 7a, b). As shown in Fig. 7c–e, the frequency, but not the amplitude, of miniature EPSCs (mEPSCs) was lower in *Neo*^*f/f*^*;Ai9* mice than that of control (*Neo*^*+/+*^*;Ai9)* mice. However, the frequency and amplitude of mIPSCs were comparable levels in the mutant neurons to that of control neurons (Fig. 7f–h). These results thus suggest that neogenin is essential for DG new born neuronal excitatory synaptic transmission.

Adult hippocampal neurogenesis is believed to be critical for prevention of depressive-like behavior and mood disorders^40^. Neogenin regulation of hippocampal neurogenesis thus leads us to speculate a role for neogenin in preventing depressive-like behavior. To test this speculation, we performed well-established mouse depressive-like behavioral paradigms, including TST (tail suspension test), FST (forced swim test), and sucrose preference test. Both control (*Neo*^*+/+*^*;Gli1-CreER*^*T2*^*;Ai9*) and *Neo*^*Gli1CreER*^-CKO mice were injected with tamoxifen for 1 month (Fig. 7i). Compared with control mice, *Neo*^*Gli1CreER*^-CKO mice displayed longer immobility time in both TST and FST tests (Fig. 7j, k). For the sucrose preference test, the consumption ratio of sucrose to water was lower in *Neo*^*Gli1CreER*^-CKO mice than that of controls (Fig. 7l), indicating reduced preference for a palatable sucrose solution. These results suggest a depressive-like behavior in *Neo*^*Gli1CreER*^-CKO mice, supporting the view for neogenin in adult hippocampal NSCs/NPCs to promote neurogenesis as well as to prevent depressive-like behavior.

## Discussion

Here, we provide evidence for neogenin’s functions in regulating adult mouse hippocampal neurogenesis, and propose a working model described in Fig. 7m. In this model, neogenin expression in NSCs/NPCs promotes their proliferation likely by increasing Gli1, a positive regulator of NSCs/NPCs proliferation^41,42^. In addition, neogenin in NSCs/NPCs prevents their astrocytic differentiation, but not neuronal differentiation. Furthermore, neogenin in immature neurons enhances dendritic outgrowth and branching, and thus elevating their excitatory neurotransmission and preventing depressive-like behavior. Taken together, these results not only identify neogenin’s unrecognized functions in adult hippocampal neurogenesis, but also implicate neogenin as a potential drug-able target for depression disorders.

Our results that neogenin is highly expressed in adult mouse hippocampal NSCs/NPCs in vivo and in vitro (Fig. 1) are in line with the reports for neogenin to be highly expressed in embryonic and adult NSCs^32,43–46^. Further supporting this view, neogenin is also detectable in Nestin^+^ and Sox2^+^ hippocampal stem cells in rats^47^. It is found that neogenin regulates neuroblast migration and terminal differentiation in the OB^26^, and controls the balance of neurons and glial cells produced in the olfactory epithelium^27^. Whereas these reports demonstrate neogenin’s functions in adult SVZ-OB neurogenesis and olfactory epithelium development, it remains unclear about neogenin’s function in adult mouse hippocampal NSCs/NPCs. Here we addressed this issue by taking advantage of various neogenin conditional knock out mice lines, including *Neo*^*Gli1CreER*^-CKO, *Neo*^*NestinCreER*^-CKO, *Neo*^*GFAPCreER*^-CKO, and *Neo*^*NexCre*^-CKO. When neogenin was selectively depleted in NSCs/NPCs (e.g., in *Neo*^*Gli1CreER*^-CKO and *Neo*^*NestinCreER*^-CKO mice), NSCs/NPCs’ proliferation and neurogenesis were impaired, but their astrogliogenesis was elevated (Figs. 2–5), demonstrating neogenin’s function in regulating adult hippocampal neurogenesis.

Neogenin regulating adult hippocampal neurogenesis appears to have three different aspects. First, neogenin promotes NSCs/NPCs’ proliferation, thus, increasing new born neurons. This is supported by our observations that the reduced tdTomato^+^ cells or DCX^+^ immature neurons in neogenin mutant DG cooperated well with the decreased proliferative NSCs/NPCs (Figs. 2, 4 and 5b), whereas the neural differentiation accessed in adult hippocampus and in NSCs culture were not affected by neogenin depletion (Figs. 3b, 5e). However, it remains unclear whether decreased proliferative NSCs/NPCs in neogenin mutant DG is due to the inhibition of proliferation speed or rate. Although more NSCs/NPCs were found to exit the cell cycle when neogenin was depleted (Fig. 4b, e), it is of great need to further explore the detailed functions of neurogenin in cell dividing. Second, neogenin suppresses astrocytic differentiation from NSCs/NPCs, thus, enhancing neurogenesis. This is based on our observations of elevated GFAP^+^ astrocytes (GFAP^+^;Td^+^/Td^+^) in neogenin mutant DG (Fig. 3f) and increased GFAP^+^ astrocytes in neogenin mutant NSCs culture (Fig. 5d). Third, neogenin enhances new born neuronal maturation by increasing dendritic outgrowth and branching and new synapse formation. Supporting this view are observations that dendritic length, branching and spines were markedly reduced, as well as glutamatergic neuro-transmission was impaired in neogenin deficient new born neurons of DG (Figs. 6, 7c).

The defective NSCs/NPCs proliferation in neogenin mutant DG appeared to be due in large to an impaired Shh signaling to Gli1 (Fig. 5). Gli1 is a GLI-Kruppel family member and sensitive to Shh activity, and expressed in both quiescent stem cells and transient-amplifying cells, but not immature neurons^35^. It is reduced in neogenin-deficient NSCs (Fig. 5h–j), and expression of Gli1 into neogenin-deficient NSCs could restore their proliferation (Fig. 5k, l). These results support the view for neogenin to promote shh signaling to Gli1, thus, regulating NSC/NPC proliferation and neurogenesis (Fig. 7m). This view is also in agreement with the reports that neogenin appears to be a direct Shh downstream regulator in neural precursor proliferation^48^; and neogenin interacts with CDO (cell adhesion molecule-related/down regulated by oncogenes), a co-receptor for Shh to induce Shh signaling to Gli1^49,50^.

Notice that we have previously reported a role for neogenin to promote neocortical astrocytic differentiation^32^. To our surprise, neogenin’s function in hippocampus appeared to be different/opposite from that in neocortex, where, it plays a negative role in hippocampal astrogliogenesis (Fig. 3f, 5d). These observations thus suggest a brain region-dependent function of neogenin in regulating astrocytic differentiation. Neogenin’s differential effects on astrogliogenesis may be resulted from its differential regulations of different signaling pathways. In neocortex, neogenin is necessary for BMP induced signaling (SMAD1/5/8 phosphorylation) and astrocytic differentiation^32^; whereas in adult hippocampus, neogenin appears to be required for shh signaling to Gli1. Thus, it is of interest to further investigate how neogenin in different brain regions participates into different signaling pathways.

The continuous generation of new neurons in adult hippocampus plays a critical role during brain development, and new-born neurons represent a distinct population of cells with enhanced synaptic plasticity^51^. The new-born neuronal dendritic development and synaptic plasticity have been implicated in mood regulation, such as depression^52–54^. In light of these observations and considering neogenin’s function in new-born neuronal maturation and function, we speculate a role for neogenin to regulate depressive-like behavior. Indeed, this appears to be the case. *Neo*^*Gli1CreER*^-CKO mice not only exhibit defective new born neuronal maturation and function, but also show depressive-like behavior. However, the relevant circuitry and molecular mechanisms underlying dendritic development remain largely unknown, it is thus of great interest to further investigate neogenin’s function in newborn neuron maturation and integration to the hippocampal circuits.

## Materials and methods

### Animals and mouse breeding

Neogenin mutant mice, generated by Bay Genomics, were kindly provided by Dr. Sue Ackerman (The Jackson Laboratory). *Neogenin*^*f/f*^ (*Neo*^*f/f*^) mice were maintained and genotyped as described previously^32^. *Nestin-Cre, Nex-Cre, Gli1-CreER*^*T2*^*, Nestin-CreER*^*T2*^*, Ai9* mice were purchased from the Jackson Laboratory. *GFAP-CreER*^*T2*^ mice were a gift from K.D. McCarthy (University of North Carolina)^55^. We crossed *Gli1-CreER*^*T2*^ mice with *Ai9* mice to get *Gli1-CreER*^*T2*^*;Ai9* mice (Supplementary Fig. S1A, B). *Neo*^*w/w*^*;Gli1-CreER*^*T2*^*;Ai9* (Control) and *Neo*^*f/f*^*;Gli1-CreER*^*T2*^*;Ai9* (*Neo*^*Gli1CreER*^-CKO) mice were generated as described in Supplementary Fig. S2A, B. *Neo*^*Nestin*^*-*CKO, *Neo*^*Nex*^*-*CKO*, Neo*^*NestinCreER*^*-*CKO, *Neo*^*GFAPCreER*^-CKO, *Neo*^*f/f*^*;Ai9* mice were generated by crossing *Neo*^*f/f*^ with *Nestin-Cre, Nex-Cre, Nestin-CreER*^*T2*^*, GFAP-CreER*^*T2*^ and *Ai9* mice, respectively. The *Ai9* mice contain a loxP-flanked STOP cassette preventing transcription of a CAG promoter-driven red fluorescent protein variant (tdTomato). Thus, tdTomato is expressed following Cre mediated recombination. All the mouse lines indicated above were maintained in C57BL/6 strain background for >6 generations. To exclude the potential interference of different sex, only male mice were examined throughout the experiments. All mouse procedures used in this study were performed in accordance with protocols approved by the Institutional Animal Care and Use Committee at Augusta University.

### Antibody and plasmids

Primary antibodies used in this project and their final concentrations were as follow: anti-β-gal (aves, 1:1000), anti-Nestin (Sigma, 1:300), anti-GFAP (Dako, 1:1000), anti-Mcm2 (BD, 1:500), anti-DCX (Santa Cruz, 1:200), anti-NeuN (Millipore, 1:1000), anti-Brdu (Accurate chemical & scientific corporation, 1:500), anti-Ki67 (Millipore, 1:200), anti-cleaved-caspase3 (CST, 1:200), anti-GFP (aves, 1:500), anti-neognin (Santa Cruz, 1:200), anti-Gli1 (abcam, 1:300), anti-GAPDH (Santa Cruz, 1:500). All the corresponding conjugated secondary antibody (1:1000) were purchased from Invitrogen. Nuclei were stained with 4′,6-diamidino-2-phenylindole (DAPI) (1:1000, Roche). d-Cre and Cre plasmids were a kind gift from Dr. Wei-Xiang Guo in University of Chinese Academy of Sciences. pBABEPuro-HA-Gli1 (#62967) plasmid was purchased from addgene.

### Drug treatment

To inducible knockout neogenin in specific cell lineage in vivo, Control and *Neo*^*Gli1CreER*^-CKO mice; *Neo*^*f/f*^ and *Neo*^*NestinCreER*^*-*CKO mice; *Neo*^*f/f*^ and *Neo*^*GFAPCreER*^-CKO mice were given indicated injections of tamoxifen based on a published paradigm^54^. Tamoxifen (Sigma) was dissolved in corn oil at 20 mg/ml. For Brdu incorporation assay, mice were given four injections of Brdu (50 mg/kg/time, 1 time/4 h) within 12 h as previously described^56^. Mice were then sacrificed and perfused for Brdu immunostaining to analyze the proliferation index.

### X-gal staining

*Neo*^*+/−*^ mice were deeply anesthetized with ketamine/xylazine (Sigma, 100/20 mg/kg, respectively, ip), and then perfused with 0.01 M phosphate buffered saline (phosphate-buffered saline (PBS), pH = 7.4) followed by 4% paraformaldehyde (PFA) in PBS. The brains were sectioned into 40 μm-thick slices with a freezing microtome, and incubated with X-gal staining solution for 12–16 h at 37 °C. After rinsed three times in PBS, the slices were counterstained with neutral red for 50 s, washed twice with ddH_2_O, and over-laid with Permount TM Mounting Medium. Finally, the slices were covered with glass coverslips for imaging and analyze.

### Stereological cell counting

For quantification of tdTomato^+^ cells, immature neuron (DCX^+^) and proliferating NSC/NPC (Brdu^+^ or Ki67^+^) densities, 40-μm thick brain sections were immunostained with RFP, DCX, Brdu and Ki67 antibodies. One in every eight serial sections starting at the beginning of hippocampus (Bregma −1.06 mm) to the end of hippocampus (Bregma −3.80 mm) were examined with a Zeiss confocal system (FM300), and each section was separated into ten *z*-plane images by a 4-μm step. About eight sections of each DG were counted, and total counts of eight examined sections were multiplied by 8 to estimate the total number of tdTomato^+^ cells per DG. To quantify the cell density (number/volume) (DCX^+^, Ki67^+^ or Brdu^+^), we firstly measured the volume (area × thickness, 40 μm) of every ninth section through the entire DG with ImageJ software, and then calculated the cell number per mm^3^. Finally, data were presented as the mean of each counted section.

### Primary culture of adult hippocampal NSCs in vitro

Neurospheres were prepared and maintained as described previously with slight modification^57,58^. Two-month-old male mice (*Neo*^*f/*f^ and *Neo*^*Nestin*^*-*CKO) were killed and then dissected DG to culture adult hippocampal NSCs. About 2 weeks later, neurospheres will be formed and then passaged for further study. All the measurements were carried out with the same number of passages.

### Histology

Two to three-month-old mice were perfused with 4% (w/v) PFA in phosphate buffer (PBS) (pH 7.4), and the dissected brains were postfixed in 2% PFA at 4 °C overnight. Coronal sections (40 μm) were washed 3 times with PBS (5 min each) and treated with blocking reagent (10% Donkey Serum + 0.5% Triton 100×) for 1 h, then incubated overnight at 4 °C with the primary antibody. Sections were washed 3 times and incubated with corresponding conjugated secondary antibody for 2 h. DAPI was used for nucleus counter staining.

### Western blot

Cultured NSCs were lysed in cell lysis buffer to get protein samples. According to the protein weight, we prefer 10% SDS-PAGE gel to separate proteins and then transfer them onto the nitrocellulose (NC) membrane. After electro-transfer, nitrocellulose membranes were blocked in western wash solution plus 5% low fat dried milk for 1 h at room temperature. Relative primary antibodies were diluted in proper concentrations to incubate NC membrane overnight at 4 °C. Membranes were washed and incubated for 1 h at room temperature with an appropriate horseradish-peroxidase-conjugated secondary antibody (1:5000, Thermo). For quantitative analysis, protein bands detected by ECL (Pierce, Rockford, IL) were scanned into pictures and analyzed using Image J software.

### qRT-PCR

Total RNA was isolated from cultured NSCs with Trizol reagent and purified RNA (5 μg) was used for cDNA synthesis using the RevertAid First Strand cDNA Synthesis Kit (Thermo Scientific). cDNA products were used for subsequent qRT-PCR (20 μl) containing the SYBR GreenER qPCR SuperMix Universal (Invitrogen). We quantified gene expression using the follow primers: Gli1, forward: 5′CTCAAACTGCCCAGCTTAACCC3′; reverse: 5′TGCGGCTGACTGTGTAAGCAGA3′; Gli2, forward: 5′ACACTGTGGAGGACTGCCTACA3′; reverse: 5′GGC ATCTCCATGCCACTGTCAT3′; Gli3, forward: 5′TCCATGGCTCTCTACCACATC3′; reverse: 5′GTGGCAGCTGAGGGAAGGAT3′; ptc1, forward: 5′AACAAAAATTCAACCAAACCTC3′; reverse: 5′TGTCTTCATTCCAGTTGATGTG3′; smo, forward: 5′TGCCACCAGAAGAACAAGCCA3′; reverse: 5′GCCTCCATTAGGTTAGTGCGG3′; neogenin, forward: 5′GAGATGGGGGACTCTACCG3′; reverse: 5′TGTCAAGCACTTCTTCGTT3′; GAPDH, forward: 5′CATCACTGCCACCCAGAAGACTG3′; reverse: 5′ATGCCAGTGAGCTTCCCGTTCAG3′. All samples were amplified in duplicate, and every experiment was repeated at least independently 3 times. The amount of amplified target cDNA was converted using the 2^-△△Ct^ method against the GAPDH to represent the relative levels in each sample.

### Production of retrovirus and virus grafting in vivo

Production of retrovirus was performed as described previously with slight modification^59^. Briefly, retroviral plasmids were transfected into cultured 293-GPG cells using polyethyleneimine, 293-GPG cells are stably transfected packaging cells, retrovirus was collected at 48, 72, and 96 h post transfection, filtered by a 0.2 μm filter, and then concentrated at 20,000 rcf for 2 h at 4 °C using a SW27 rotor (Beckman). Removed the supernant and resuspended the retrovirus in 100 μl PBS.

For in vivo retrovirus grafting, 2-month old mice were anesthetized with ketamine/xylazine (Sigma, 100/20 mg/kg, respectively, ip), 1 μl d-Cre (an inactive Cre, as a control) retrovirus was injected into the left DG of brain and 1 μl Cre retrovirus was injected into the other side. The injected coordinates were relative to bregma, caudal: −2.0 mm; lateral: + /−1.6 mm; ventral: −2.0 mm.

### Retrovirus infection in cultured NSCs in vitro

Neurospheres from *Neo*^*f/*f^ and *Neo*^*Nestin*^*-*CKO mice were digested into single cells and seeded at 1 × 10^5^ cells/ml. Subsequently, concentrated Gli1-HA retroviruses were added into proliferation medium to infect NSCs of *Neo*^*Nestin*^*-*CKO mice. 3 days later, immunostaining of HA tag indicated the expressing efficiency of Gli1 in cultured NSCs in vitro.

### Electrophysiological recording

Hippocampal slices were prepared as described previously^60^. mEPSCs were recorded at holding potential of −70 mV in the presence of 20 μM BMI and 1 μM TTX, with the pipette solution containing (in mM): 125 K-gluconate, 5 KCl, 10 Hepes, 0.2 EGTA, 1 MgCl2, 4 Mg-ATP, 0.3 Na-GTP and 10 phosphocreatine (pH 7.40, 285 mOsm). To record miniature IPSCs (mIPSCs), neurons were held at −70 mV in the presence of 20 μM CNQX, 100 μM DL-AP5 and 1 μM TTX. Recording pipettes were filled with the solution contained (in mM): 140 CsCl, 10 Hepes, 0.2 EGTA, 1 MgCl_2_, 4 Mg-ATP, 0.3 Na-GTP, 10 phosphocreatine and 5 QX314 (pH 7.4, 285 mOsm). In all experiments, series resistance was controlled below 20 MΩ and not compensated. Cells would be rejected if membrane potentials were more positive than −60 mV; or if series resistance fluctuated more than 20% of initial values. All recordings were done at 32–34 °C. Data were filtered at 1 kHz and sampled at 10 kHz.

### Behavioral analysis

Two to three-month old mice (male) were housed together and used for behavioral studies. The TST and FST were performed as described previously with slight modification^61,62^. The last 4-min of a 6-min test were analyzed and the immobility time was measured directly. The sucrose preference test was carried out using a two bottle choice procedure. Single housed mice were habituated to drink 2% (wt/vol) sucrose solution (dissolved in water) for 3 days, then mice were given access to the two preweighed bottles, one containing water and the other containing 2% sucrose solution. We changed bottle positions every day and assessed water and sucrose solution consumption daily for 4 days. The consuming ratio of sucrose over water was used for measuring the sucrose preference.

### Statistical analysis

All results presented in this study were from at least three independent experiments. Statistical analyses were performed using Student’s *t* test, or one-way ANOVA followed by a Bonferroni post hoc analysis. All data were presented as mean ± SEM and described in the figure legends. A value of *P* < 0.05 was considered to be significant. All graphics were prepared using GraphPad Prism 5 software.

## Electronic supplementary material

Supplementary Figures

FigureS1

FigureS2

FigureS3

FigureS4

FigureS5

FigureS6
