# Supplementary material for: Neogenin, a regulator of adult hippocampal neurogenesis, prevents depressive-like behavior
Source: Cell Death Dis. 2018 Jan 8;9(1):8. doi: 10.1038/s41419-017-0019-2 (PMC5849041; doi:10.1038/s41419-017-0019-2)
Supplement: Supplementary file 1 — Supplementary Figures [file 41419_2017_19_MOESM1_ESM.docx]

**Supplementary Figure Legends**

**Figure S1. Tamoxifen-induction of tdTomato expression in Gli1-positive cells and their progenies in *Gli1-CreER^T2^;Ai9* mice. (A)** *Gli1-CreER^T2^;Ai9* mice were generated by crossing *Gli1-CreER^T2^* with *Ai9* mice. **(B)** Genotyping of *Gli1-CreER^T2^;Ai9* mice. **(C)** Immunostaining analysis of tdTomato in *Gli1-CreER^T2^;Ai9* mice after tamoxifen injection for 7 days. Scale bars = 100μm. **(D-G)** Double immunostaining of tdTomato with indicated cell markers: Nestin (D) or GFAP (E) in SGZ for radial glial-like stem cells; Mcm2 (F) for transient amplifying cells, and DCX (G) for immature neurons. Scale bars = 10μm. **(H)** Model of neurogenesis and astrogliogenesis in adult hippocampus.

**Figure S2. Strategy for generation of inducible neogenin conditional knockout (CKO) mice. (A)** Generation of *Neo^+/+^;Gli1creER;Ai9* (control) and *Neo^f/f^;Gli1creER;Ai9* (*Neo^Gli1CreER^*-CKO) mice. **(B)** Genotyping of control and *Neo^Gli1CreER^*-CKO mice. **(C)** Schematic diagram of tamoxifen treatment for indicated time. **(D)** Co-immunostaining of tdTomato (red) with neogenin (green) in hippocampal DG of control and *Neo^Gli1CreER^*-CKO mice. Scale bars: 50μm in D and 10μm in selected region. **(E)** Quantification of relative neogenin fluorescent intensity in DG tdTomato^+^ cells of control and *Neo^Gli1CreER^*-CKO mice. (n≥300 cells). ****P* < 0.001; Student’s *t*-test. Data are presented as the mean ± SEM.

**Figure S3. No increased apoptosis in neogenin depleted hippocampus. (A)** Immunostaining of cleaved-caspase3 (c-caspase3) in hippocampus of control and *Neo^Gli1CreER^*-CKO mice. Scale bars = 100μm. **(B)** Quantitative analysis of the number of c-caspase3 positive cells per DG. **(C)** Western blot analysis of c-caspase3 expression in hippocampus of control and *Neo^Gli1CreER^*-CKO mice. **(D)** Quantification of the relative c-caspase3 protein levels in C. Data are presented as the mean ± SEM. (n=3 per genotype). ns= no significant difference. Student’s *t*-test.

**Figure S4. Reduced hippocampal neurogenesis in *Neo^NestinCreER^*-CKO mice, but not in *Neo^GFAPCreER^*-CKO or *Neo^Nex^*-CKO mice. (A)** Schematic diagram of tamoxifen treatment for indicated time. **(B, E, H)** Immunostaining of DCX (white) in hipoocampus of *Neo^f/f^*, *Neo^NestinCreER^*-CKO, *Neo^GFAPCreER^*-CKO, and *Neo^Nex^*-CKO mice. Scale bars = 100μm. **(C, F, I)** Quantitative analyses of DCX^+^ cell density in B, E and H (n=3 for each genotype, normalized to *Neo^f/f^* mice). **(D, G, J)** Quantitative analyses of relative process length of DCX^+^ cells in B, E and H. Data are presented as the mean ± SEM (n = 3 per genotype). *p < 0.05; **p < 0.01. Student’s *t*-test.

**Figure S5. Impaired growth of cultured neurospheres from *Neo^Nestin^*-CKO mice. (A)** Western blot analysis of neogenin expression in hippocampal NSCs from *Neo^f/f^* and *Neo^Nestin^*-CKO mice (n=3 per genotype). **(B)** Representative images of cultured hippocampal neurospheres from *Neo^f/f^* and *Neo^Nestin^*-CKO mice. Scale bars = 100μm. **(C)** Quantitative analysis of the neurospheres’ size (diameter) from *Neo^f/f^* and *Neo^Nestin^*-CKO mice. Data are mean ± SEM. (n ≥ 200 neurospheres). **P* < 0.05. Student’s *t*-test.

**Figure S6. Impaired dendritic development in DG of *Neo^Gli1CreER^*-CKO mice treated with tamoxifen. (A)** Diagram of tamoxifen treatment for 1 month in control and *Neo^Gli1CreER^*-CKO mice. **(B)** Double immunostaining of tdTomato (red) and NeuN (green). Scale bars = 50μm. **(C)** Tracing of representative new-born neurons in the DG of control and *Neo^Gli1CreER^*-CKO mice. **(D)** Sholl analysis of the dendritic complexity in C by ImageJ software. (n ≥ 50 cells). **(E)** Quantification of dendritic total length. (n ≥ 50 cells). **(F)** Quantification of branch number. (n ≥ 50 cells). **(G)** Representative spine images of 1-month old neurons from control and *Neo^Gli1CreER^*-CKO mice. Scale bars = 5μm. **(H)** Quantitative analysis of the number of spines in control and *Neo^Gli1CreER^*-CKO mice. **(I)** Quantitative analysis of different types of spine densities in control and *Neo^Gli1CreER^*-CKO mice. Data are mean ± SEM. (n ≥ 30 cells). **P* < 0.05; ***P* < 0.01. Student’s *t*-test.
