# Supplementary figures and images for: Neogenin, a regulator of adult hippocampal neurogenesis, prevents depressive-like behavior
Source: Cell Death Dis. 2018 Jan 8;9(1):8. doi: 10.1038/s41419-017-0019-2 (PMC5849041; doi:10.1038/s41419-017-0019-2)

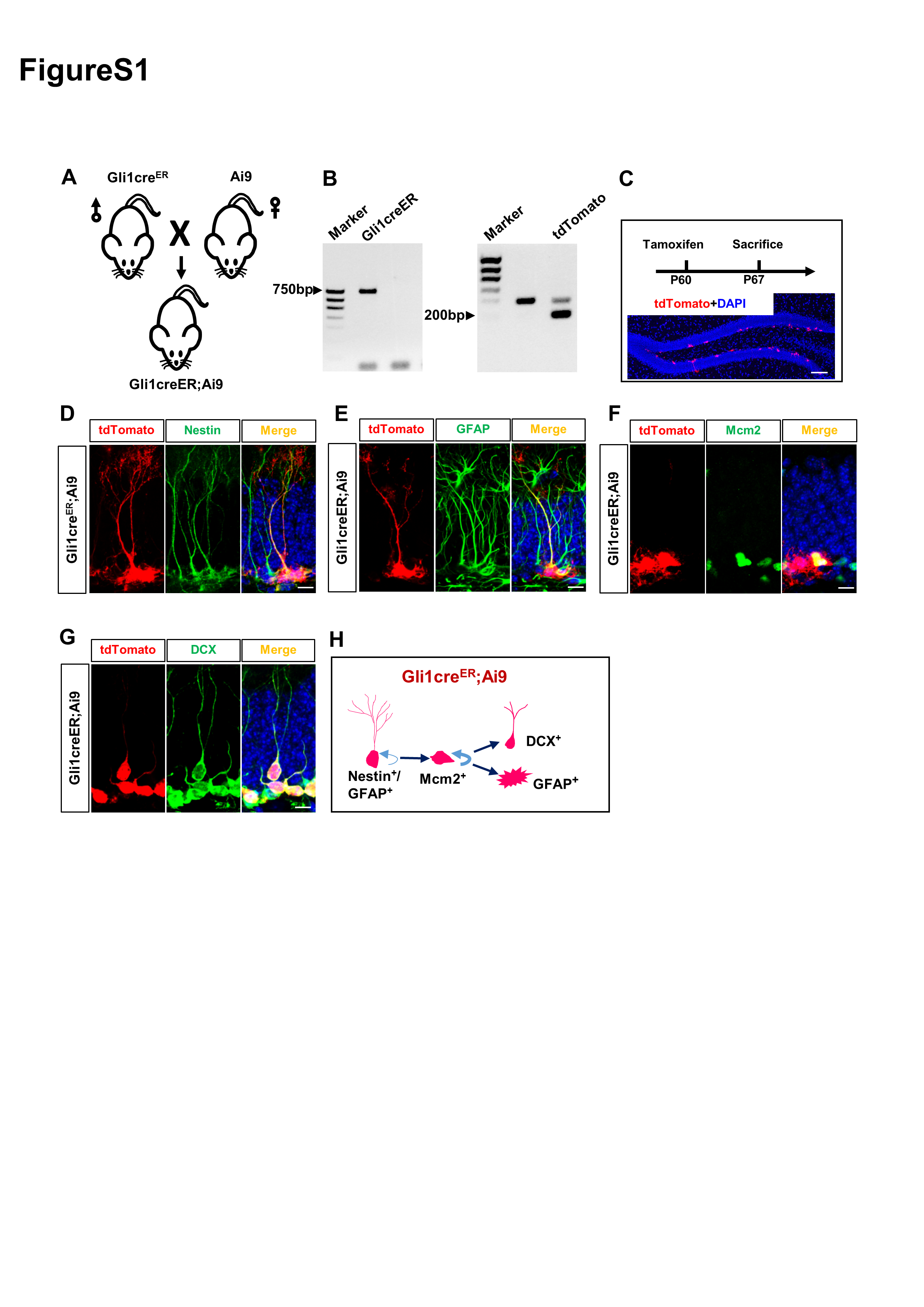

Supplement: Supplementary file 2 — FigureS1 [file 41419_2017_19_MOESM2_ESM.tif]

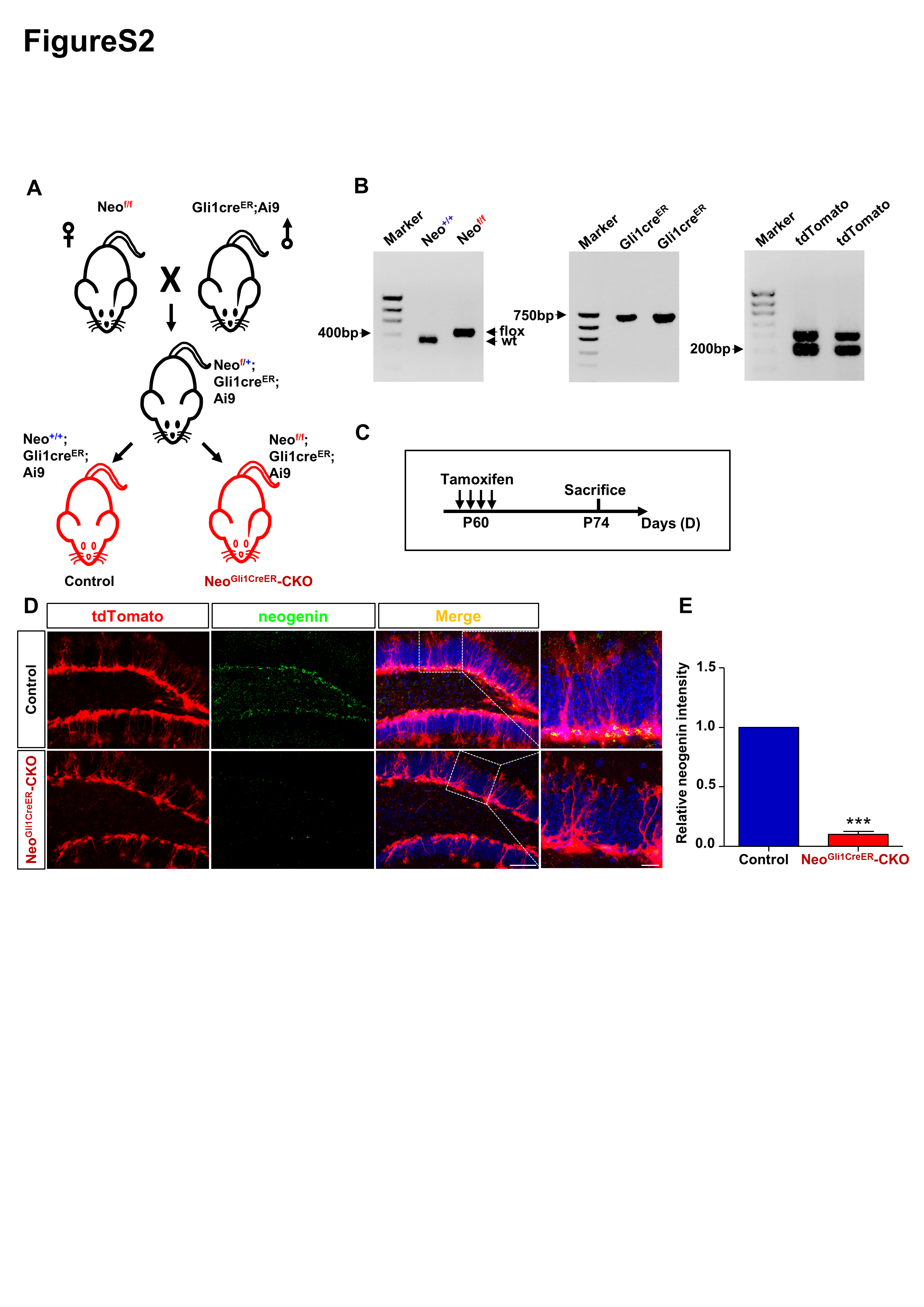

Supplement: Supplementary file 3 — FigureS2 [file 41419_2017_19_MOESM3_ESM.tif]

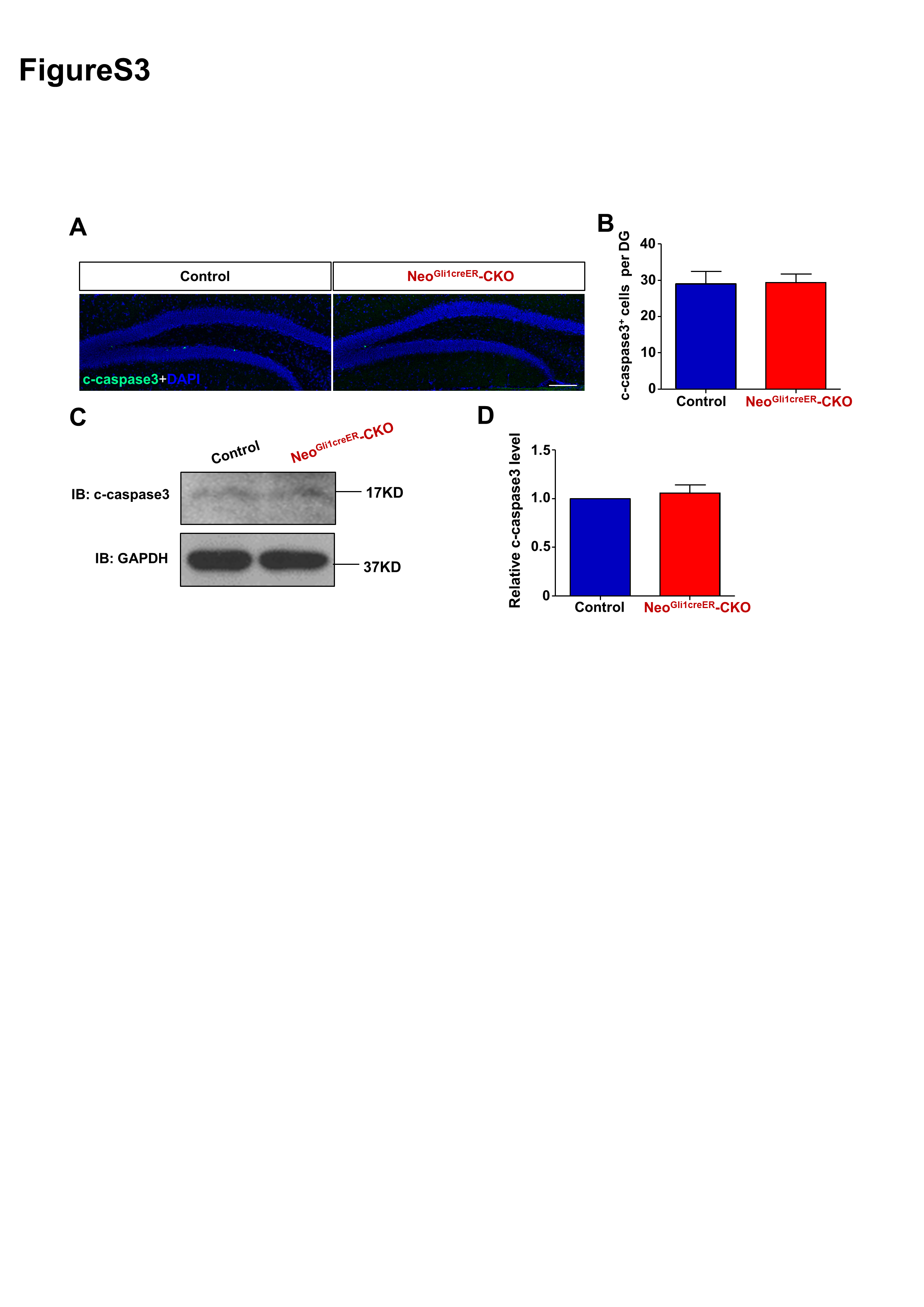

Supplement: Supplementary file 4 — FigureS3 [file 41419_2017_19_MOESM4_ESM.tif]

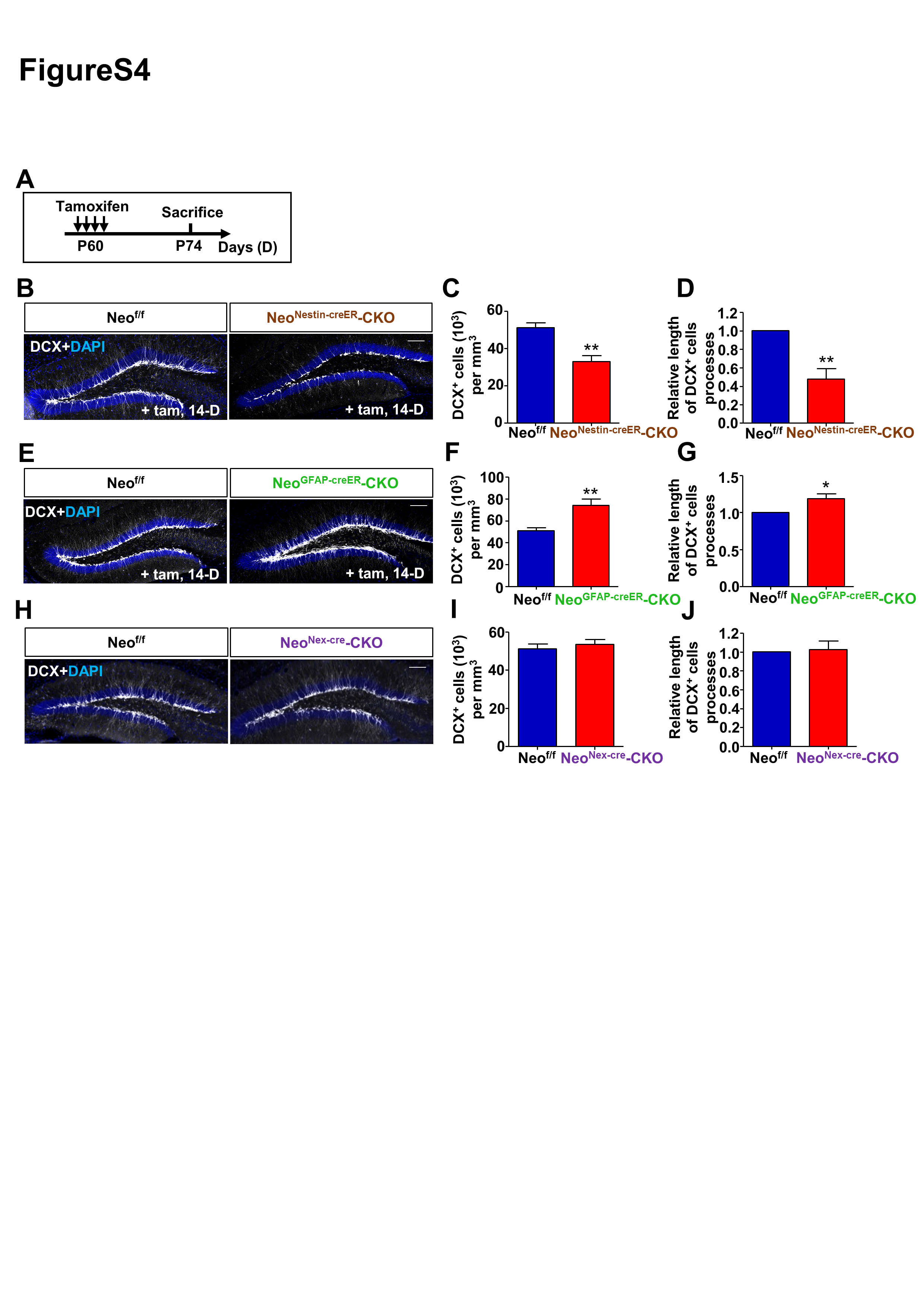

Supplement: Supplementary file 5 — FigureS4 [file 41419_2017_19_MOESM5_ESM.tif]

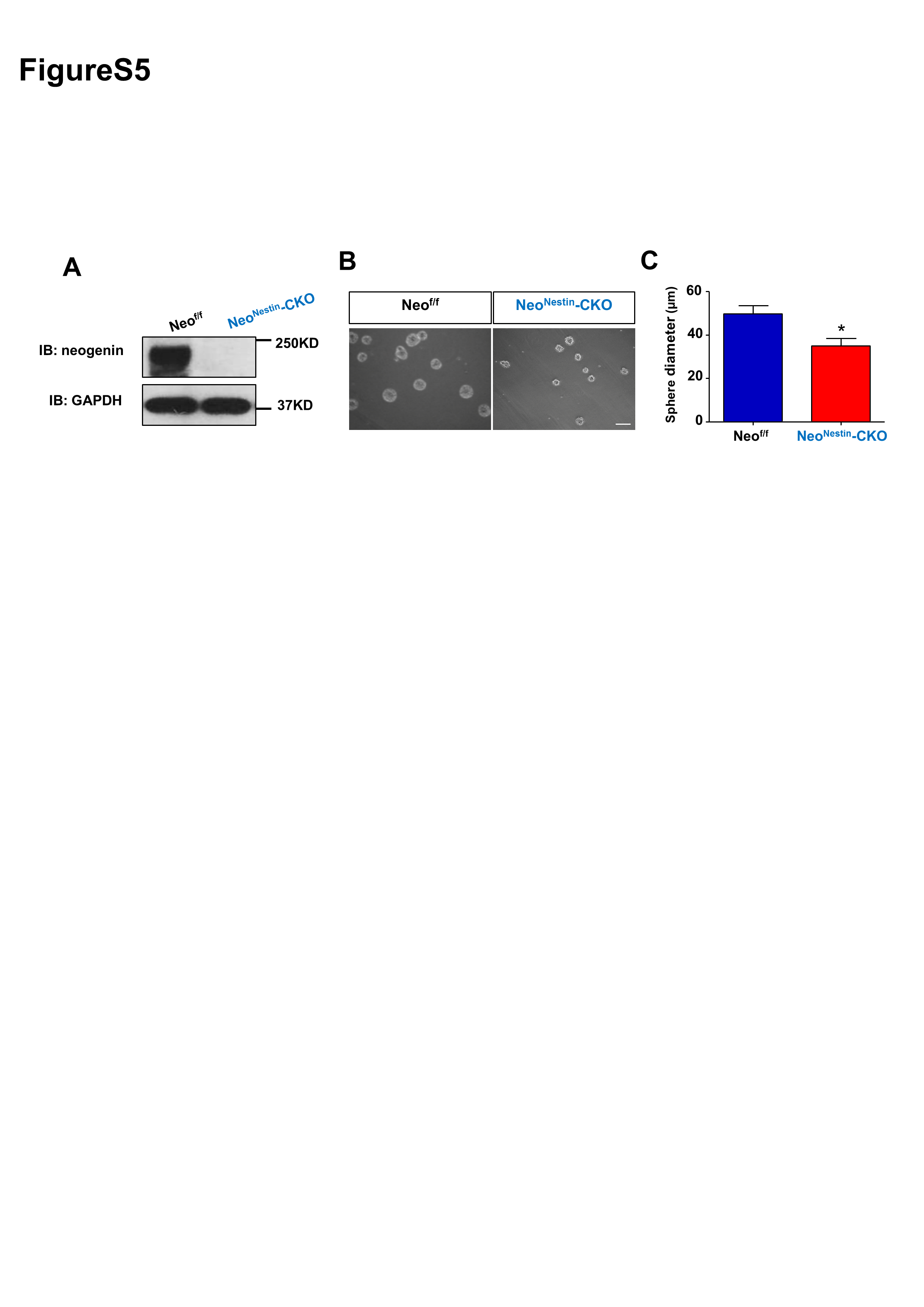

Supplement: Supplementary file 6 — FigureS5 [file 41419_2017_19_MOESM6_ESM.tif]

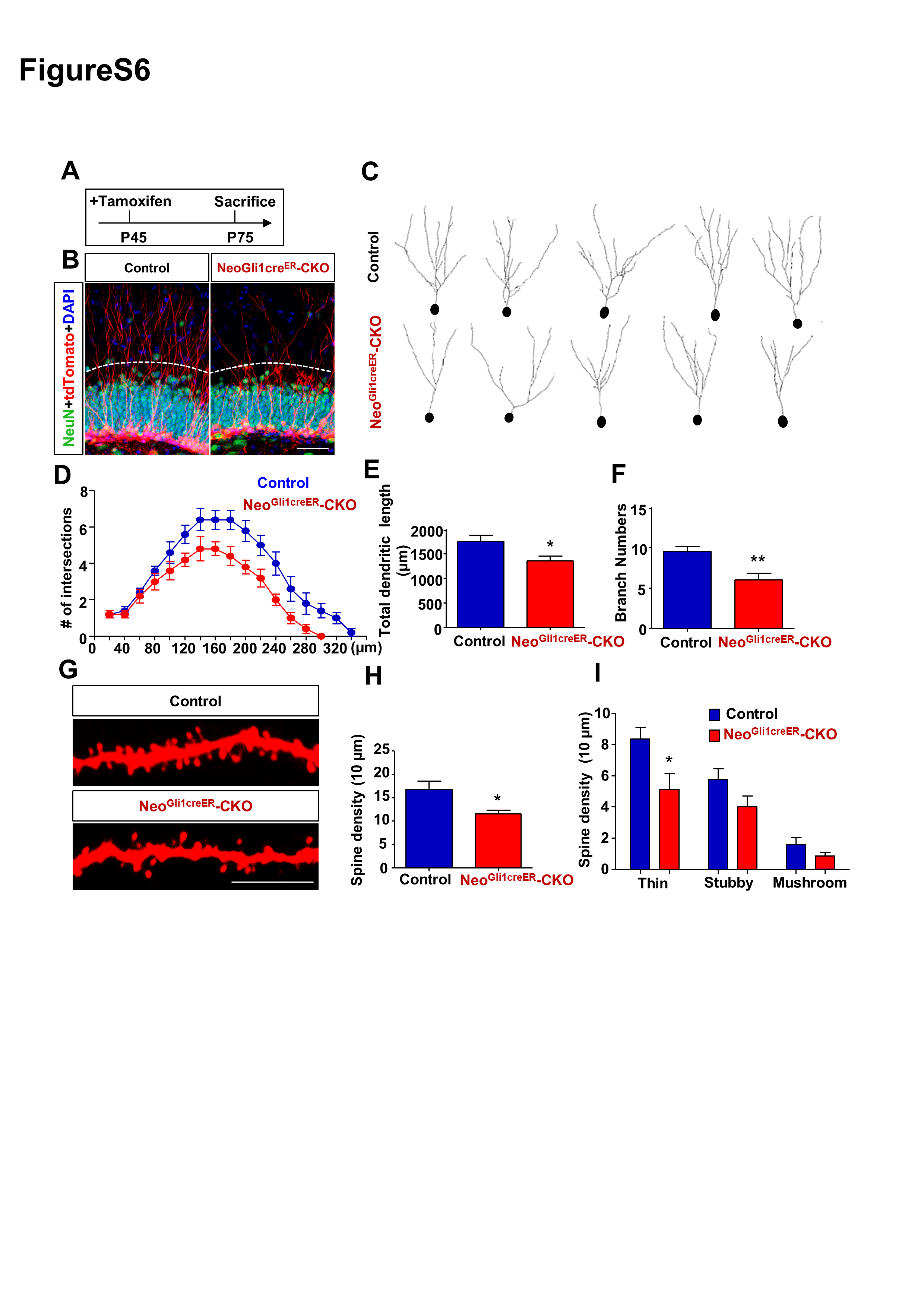

Supplement: Supplementary file 7 — FigureS6 [file 41419_2017_19_MOESM7_ESM.tif]
